# *Nlrp3* Deficiency Does Not Substantially Affect Femoral Fracture Healing in Mice

**DOI:** 10.3390/ijms252111788

**Published:** 2024-11-02

**Authors:** Maximilian M. Menger, Rouven Speicher, Sandra Hans, Tina Histing, Moses K. D. El Kayali, Sabrina Ehnert, Michael D. Menger, Emmanuel Ampofo, Selina Wrublewsky, Matthias W. Laschke

**Affiliations:** 1 Department of Trauma and Reconstructive Surgery, BG Trauma Center Tuebingen, Eberhard Karls University Tuebingen, 72076 Tuebingen, Germany; thisting@bgu-tuebingen.de (T.H.); 2 Institute for Clinical and Experimental Surgery, Saarland University, 66421 Homburg, Germany; rouven.speicher@web.de (R.S.); sandra.hans@uks.eu (S.H.); m.elkayali@yahoo.de (M.K.D.E.K.); michael.menger@uks.eu (M.D.M.); emmanuel.ampofo@uks.eu (E.A.); selina.wrublewsky@uks.eu (S.W.); matthias.laschke@uks.eu (M.W.L.); 3 Department of Trauma and Reconstructive Surgery, BG Trauma Center Tuebingen, Siegfried Weller Institute for Trauma Research, Eberhard Karls University Tuebingen, 72076 Tuebingen, Germany； sehnert@bgu-tuebingen.de

**Keywords:** NLRP3, VEGF, inflammasome, AIM2, fracture healing, bone regeneration, angiogenesis

## Abstract

Inflammation has been recognized as major factor for successful bone regeneration. On the other hand, a prolonged or overshooting inflammatory response can also cause fracture healing failure. The nucleotide-binding oligomerization domain (NOD)-like receptor protein (NLRP)3 inflammasome plays a crucial role in inflammatory cytokine production. However, its role during fracture repair remains elusive. We investigated the effects of *Nlrp3* deficiency on the healing of closed femoral fractures in *Nlrp3*^−/−^ and wildtype mice. The callus tissue was analyzed by means of X-ray, biomechanics, µCT and histology, as well as immunohistochemistry and Western blotting at 2 and 5 weeks after surgery. We found a significantly reduced trabecular thickness at 2 weeks after fracture in the *Nlrp3*^−/−^ mice when compared to the wildtype animals. However, the amount of bone tissue did not differ between the two groups. Additional immunohistochemical analyses showed a reduced number of CD68-positive macrophages within the callus tissue of the *Nlrp3*^−/−^ mice at 2 weeks after fracture, whereas the number of myeloperoxidase (MPO)-positive granulocytes was increased. Moreover, we detected a significantly lower expression of vascular endothelial growth factor (VEGF) and a reduced number of microvessels in the *Nlrp3*^−/−^ mice. The expression of the absent in melanoma (AIM)2 inflammasome was increased more than 2-fold in the *Nlrp3*^−/−^ mice, whereas the expression of the pro-inflammatory cytokines interleukin (IL)-1β and IL-18 was not affected. Our results demonstrate that *Nlrp3* deficiency does not markedly affect femoral fracture healing in mice. This is most likely due to the unaltered expression of pro-inflammatory cytokines and pro-osteogenic growth factors.

## 1. Introduction

Fracture healing has been of great interest in preclinical and clinical research during the last decades. Accordingly, there is a steadily increasing amount of knowledge about the molecular and cellular basis of bone regeneration. However, the pathophysiological mechanisms of fracture healing failure are still not satisfactorily clarified, leading to a non-union rate of up to 10% [1]. Non-union formation represents a major complication of trauma and orthopedic surgery, which is often associated with massive pain, loss of function of the affected limb and extensive revision surgeries [2,3]. The resulting prolonged rehabilitation process additionally places a significant economic burden on the health care system [4].

Many factors contribute to successful bone regeneration. Interestingly, inflammation has been proven to be of major importance. In fact, the inflammatory phase is essential for fracture repair by directing mesenchymal stem cells to the fracture site and providing the callus tissue with vital pro-osteogenic and pro-angiogenic progenitor cells [5,6]. An uncontrolled and overshooting inflammatory response at the fracture site, on the other hand, can impair the process of bone regeneration. This phenomenon is particularly evident in the aged or individuals suffering from comorbidities, such as diabetes. These patients exhibit elevated levels of circulating pro-inflammatory cytokines leading to a chronic status of inflammation and, eventually, a higher rate of non-union formation [7,8].

The nucleotide-binding oligomerization domain (NOD)-like receptor protein (NLRP) 3 inflammasome plays a crucial role in inflammatory cytokine production [9]. NLRP3 is a multi-protein complex, which consists of the sensor NLRP3, caspase-1 and the apoptosis-associated speck-like protein containing a CARD adapter protein, resulting in the maturation of the precursor forms of the pro-inflammatory cytokines interleukin (IL)-1β and IL-18 [10,11]. It has already been demonstrated that *Nlrp3* deficiency leads to impaired skeletal development [12]. However, its role in fracture healing remains elusive. Therefore, we herein investigate the effects of *Nlrp3* deficiency on the process of fracture healing in mice.

## 2. Results

### 2.1. X-Ray

**Figure 1:**
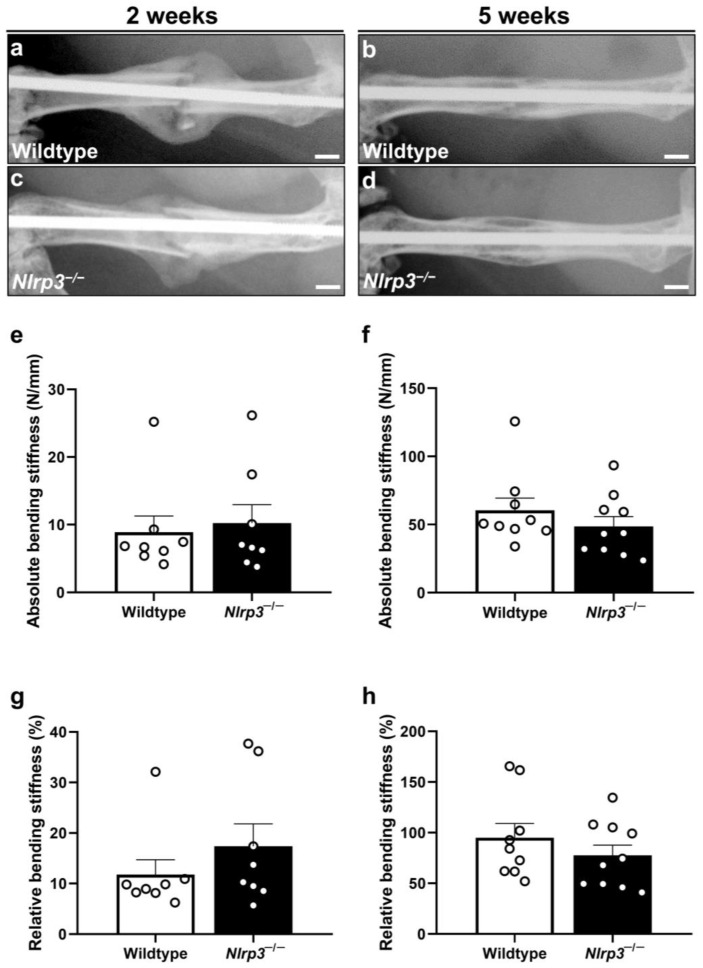
(**a**–**d**) Representative X-rays of fractured mouse femora stabilized by an intramedullary screw in wildtype (**a**,**b**) and *Nlrp3*^−/−^ mice (**c**,**d**) at 2 (**a**,**c**) and 5 weeks (**b**,**d**) after fracture. Scale bars: 1 mm. (**e**–**h**) Biomechanical analyses of absolute (**e**,**f**) and relative (**g**,**h**) bending stiffness in wildtype (white bars, *n* = 8) and *Nlrp3*^−/−^ mice (black bars, *n* = 9–10) at 2 (**e**,**g**) and 5 weeks (**f**,**h**) after fracture. Data are given as absolute values (N/mm) and as percent of contralateral, non-fractured femora (%) (mean ± SEM).

The performed X-rays demonstrated typical signs of endochondral fracture healing and bone remodeling in both groups (Figure 1a–d). These included abundant callus formation at 2 weeks and osseus bridging with callus remodeling at 5 weeks after surgery in the young adult and aged mice. Fracture or implant dislocation was not observed in the wildtype or *Nlrp3*^−/−^ mice.

### 2.2. Biomechanics

Interestingly, the biomechanical analyses revealed no significant differences in the absolute bending stiffness of the fractured femora between the two groups at 2 (*p* = 0.878) and 5 weeks (*p* = 0.206). Notably, the relative bending stiffness also showed no significant difference between the knockout and wildtype animals (2 weeks: *p* = 0.279; 5 weeks: *p* = 0.391) (Figure 1e–h). However, the bending stiffness of the unfractured femora in the *Nlrp3*^−/−^ mice was significantly reduced when compared to the wildtype animals (69.4 ± 2.7 N/mm vs. 61.1 ± 1.8 N/mm; (*p* = 0.014)).

### 2.3. µCT

**Figure 2:**
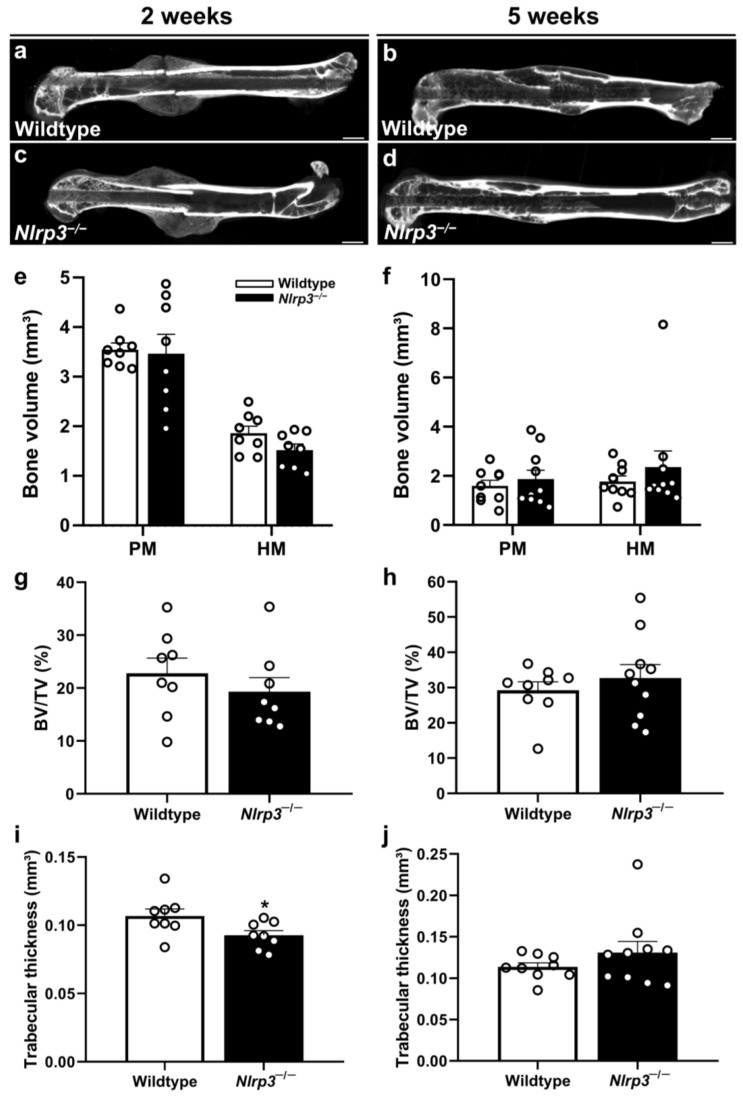
(**a**–**d**) Representative µCT images of femora in wildtype (**a**,**b**) and *Nlrp3*^−/−^ mice (**c**,**d**) at 2 (**a**,**c**) and 5 weeks (**b**,**d**) after fracture. Scale bars: 1 mm. (**e**–**j**) µCT analysis of poorly mineralized (PM) and highly mineralized (HM) bone volume (**e**,**f**), BV/TV (**g**,**h**) and trabecular thickness (**i**,**j**) in wildtype (white bars, *n* = 8) and *Nlrp3*^−/−^ mice (black bars, *n* = 9–10) at 2 (**e**,**g**,**i**) and 5 weeks (**f**,**h**,**j**) after fracture (mean ± SEM). * *p* < 0.05 vs. wildtype.

The µCT analysis showed no significant difference in the amount of poorly (2 weeks: *p* = 0.878; 5 weeks: *p* = 0.531) and highly mineralized bone volume (BV) (2 weeks: 0.095; 5 weeks: *p* = 0.775), as well as in the bone volume fraction (BV/total volume (TV)) (2 weeks: *p* = 0.391; 5 weeks: *p* = 0.467), in the *Nlrp3*^−/−^ mice when compared to the wildtype animals (Figure 2a–h). However, we detected a significantly reduced trabecular thickness in the *Nlrp3*^−/−^ mice when compared to the wildtype animals at 2 weeks after fracture (*p* = 0.037) (Figure 2i). At 5 weeks, the trabecular thickness did not differ anymore between the two groups (*p* = 0.438) (Figure 2j).

### 2.4. Histology and Histomorphometry

**Figure 3:**
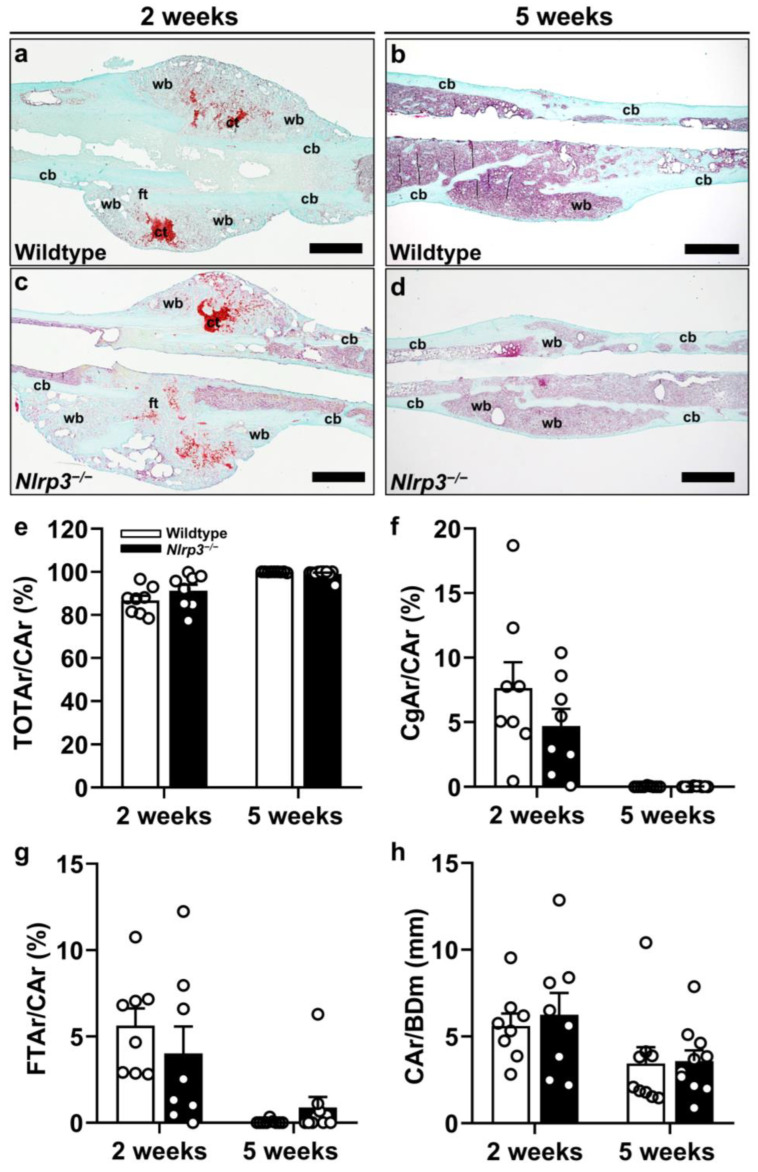
(**a**–**h**) Representative histological images of Safranin-O-stained femora of wildtype (**a**,**b**) and *Nlrp3*^−/−^ mice (**c**,**d**) at 2 (**a**,**c**) and 5 weeks (**b**,**d**) after fracture. Fibrous tissue (ft), cartilaginous tissue (ct), woven bone (wb) and cortical bone (cb) are indicated. Scale bars: 1 mm. (**e**–**h**) Histological analysis of bone (total osseous tissue) callus area/total callus area (TOTAr/CAr) (**e**), cartilaginous callus area/total callus area (CgAr/CAr) (**f**), fibrous tissue callus area/total callus area (FTAr/CAr) (**g**) and CAr/BDm (**h**) in wildtype (white bars, *n* = 8) and *Nlrp3*^−/−^mice (black bars, *n* = 9–10) at 2 and 5 weeks after fracture (mean ± SEM).

The Safranin-O staining showed typical signs of endochondral fracture healing and subsequent osseous bridging in both study groups (Figure 3a–d). These included cartilaginous tissue formation with subsequent replacement with novel bone tissue and subsequent callus remodeling at later time points. The histomorphometric analysis of the Safranin-O staining demonstrated no significant differences in the callus composition or the ratio of total callus area (bone, cartilaginous and fibrous callus area) to femoral bone diameter (cortical width plus marrow diameter) at the fracture gap (CAr/BDm) (2 weeks: *p* = 0.667; 5 weeks: *p* = 0.488) between the *Nlrp3*^−/−-^ and wildtype mice (Figure 3e–h). In fact, our results demonstrated no significant differences in the ratio of osseous (2 weeks: *p* = 0.225; 5 weeks: *p* = 0.102), cartilaginous (2 weeks: *p* = 0.238 5 weeks: *p* = 0.396) and fibrous tissue (2 weeks: *p* = 0.399; 5 weeks: *p* = 0.109) (Figure 3e–g).

**Figure 4:**
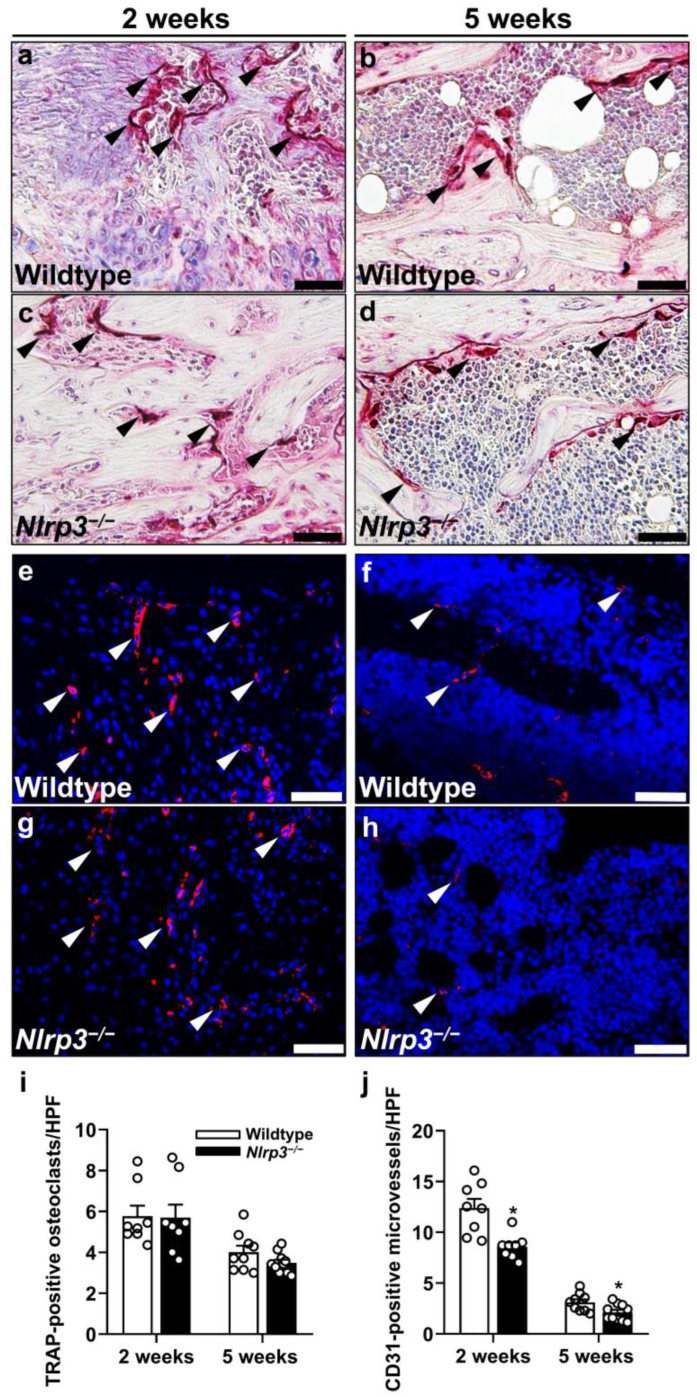
(**a**–**d**) Representative histological images of TRAP-positive osteoclasts (arrowheads) within callus tissue of wildtype (**a**,**b**) and *Nlrp3*^−/−^ mice (**c**,**d**) at 2 (**a**,**c**) and 5 weeks (**b**,**d**) after fracture. Scale bars 50 µm. (**e**–**h**) Representative immunohistochemical images of CD31-positive microvessels (arrowheads) within the callus tissue of wildtype (**e**,**f**) and *Nlrp3*^−/−^ mice (**g**,**h**) at 2 (**e**,**g**) and 5 weeks (**f**,**h**) after fracture. Scale bars: 50 µm. Histological analysis of TRAP-positive osteoclasts/HPF (**i**) and immunohistochemical analysis of CD31-positive microvessels/HPF (**j**) within callus tissue of wildtype (white bars, *n* = 8) and *Nlrp3*^−/−^ mice (black bars, *n* = 9–10) at 2 and 5 weeks after fracture (mean ± SEM). * *p* < 0.05 vs. wildtype.

In line with these findings, the assessment of the number of tartrate-resistant acid phosphatase (TRAP)-positive osteoclasts within the callus tissue revealed no significant difference between the two groups throughout the observation period (2 weeks: *p* = 0.878; 5 weeks: *p* = 0.189) (Figure 4a–d,i).

### 2.5. Immunohistochemistry

The immunohistochemical analysis of vascularization showed a significantly decreased number of CD31-positive microvessels at 2 (*p* = 0.001) and 5 weeks (*p* = 0.022) after fracture within the callus tissue of the *Nlrp3*^−/−^ mice when compared to the wildtype animals (Figure 4e–h,j).

**Figure 5:**
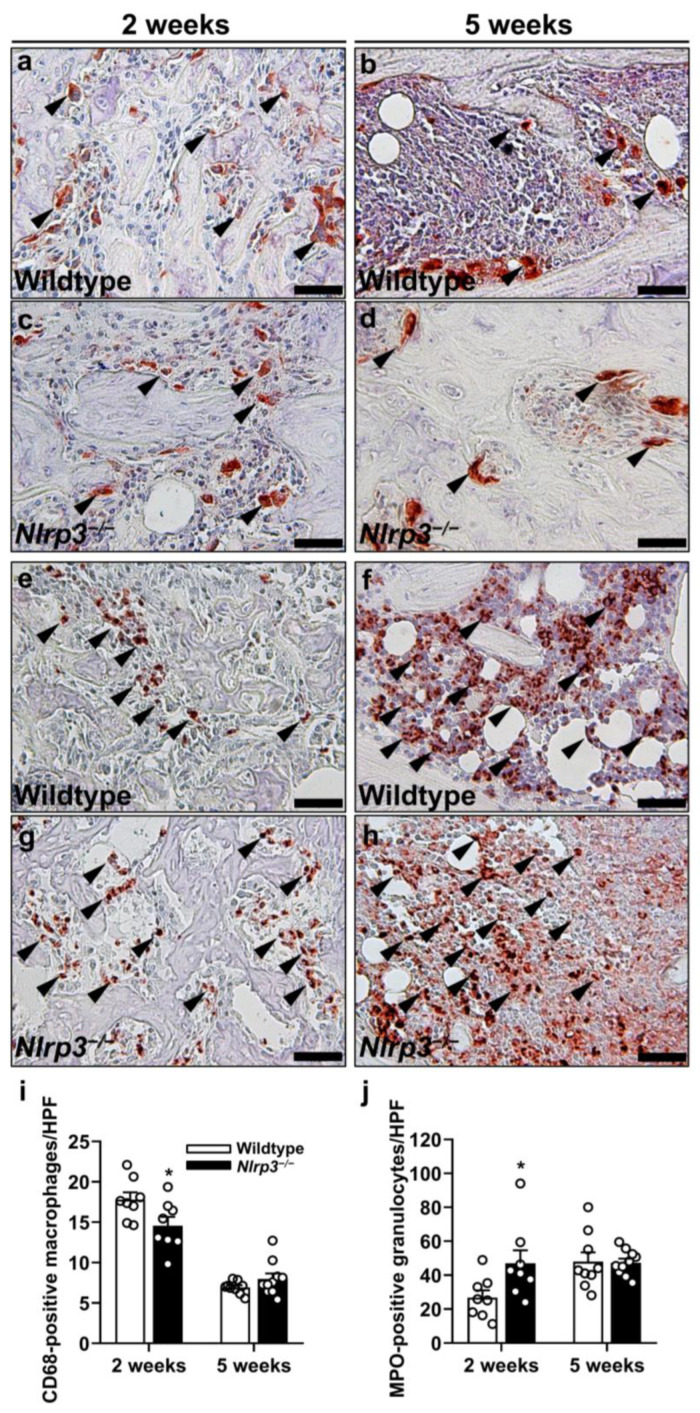
(**a**–**d**) Representative immunohistochemical images of CD68-positive macrophages (arrowheads) within callus tissue of wildtype (**a**,**b**) and *Nlrp3*^−/−^ mice (**c**,**d**) at 2 (**a**,**c**) and 5 weeks (**b**,**d**) after fracture. Scale bars: 50 µm. (**e**–**h**) Representative immunohistochemical images of MPO-positive granulocytes (arrowheads) within callus tissue of wildtype (**e**,**f**) and *Nlrp3*^−/−^ mice (**g**,**h**) at 2 (**e**,**g**) and 5 weeks (**f**,**h**) after fracture. Scale bars: 50 µm. Immunohistochemical analysis of CD68-positive macrophages/HPF (**i**) and MPO-positive granulocytes/HPF (**j**) within callus tissue of wildtype (white bars, *n* = 8) and *Nlrp3*^−/−^ mice (black bars, *n* = 9–10) at 2 and 5 weeks after fracture (mean ± SEM). * *p* < 0.05 vs. wildtype.

Moreover, we detected a significantly reduced number of CD68-positive macrophages at 2 weeks after fracture in the *Nlrp3*^−/−^ mice when compared to the wildtype animals (*p* = 0.038), whereas the number of myeloperoxidase (MPO)-positive granulocytes was significantly increased (*p* = 0.038) (Figure 5a–j). Of note, at 5 weeks after fracture the number of macrophages (*p* = 0.205) and granulocytes (*p* = 0.917) did not significantly differ anymore between the two groups (Figure 5a–j).

### 2.6. Western Blot

**Figure 6:**
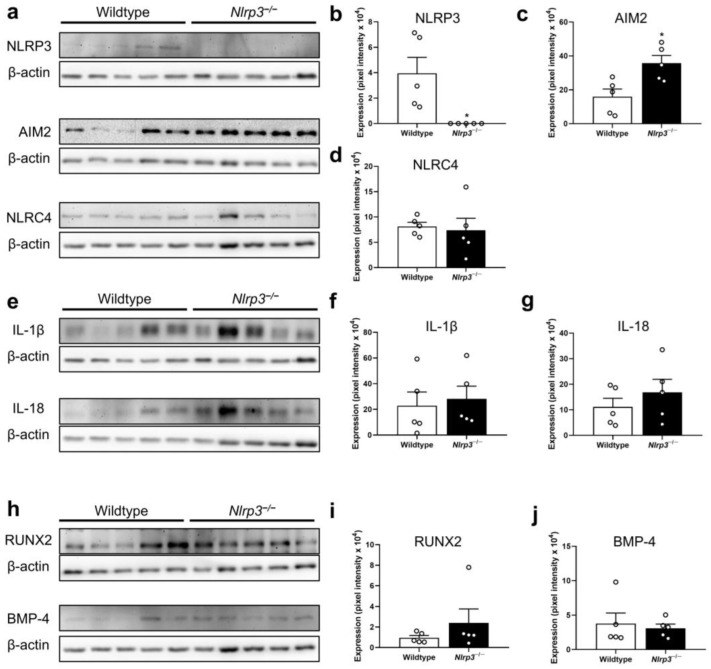
(**a**) Representative Western blots of NLRP3, AIM2, NLRC4 and β-actin expression within callus tissue of wildtype and *Nlrp3*^−/−^ mice at 2 weeks after fracture. (**b**–**d**) Expression of NLRP3 (**b**), AIM2 (**c**) and NLRC4 (**d**) within callus tissue of wildtype (white bars, *n* = 5) and *Nlrp3*^−/−^ mice (black bars, *n* = 5) (mean ± SEM). * *p* < 0.05 vs. wildtype. (**e**) Representative Western blots of IL-1β, IL-18 and β-actin expression within callus tissue of wildtype and *Nlrp3*^−/−^ mice at 2 weeks after fracture. (**f**,**g**) Expression of IL-1β (**f**) and IL-18 (**g**) within callus tissue of wildtype (white bars, *n* = 5) and *Nlrp3*^−/−^ mice (black bars, *n* = 5) (mean ± SEM). (**h**) Representative Western blots of RUNX2, BMP-4 and β-actin expression within callus tissue of wildtype and *Nlrp3*^−/−^ mice at 2 weeks after fracture. (**f**,**g**) Expression of RUNX2 (**i**) and BMP-4 (**j**) within callus tissue of wildtype (white bars, *n* = 5) and *Nlrp3*^−/−^ mice (black bars, *n* = 5) (mean ± SEM).

**Figure 7:**
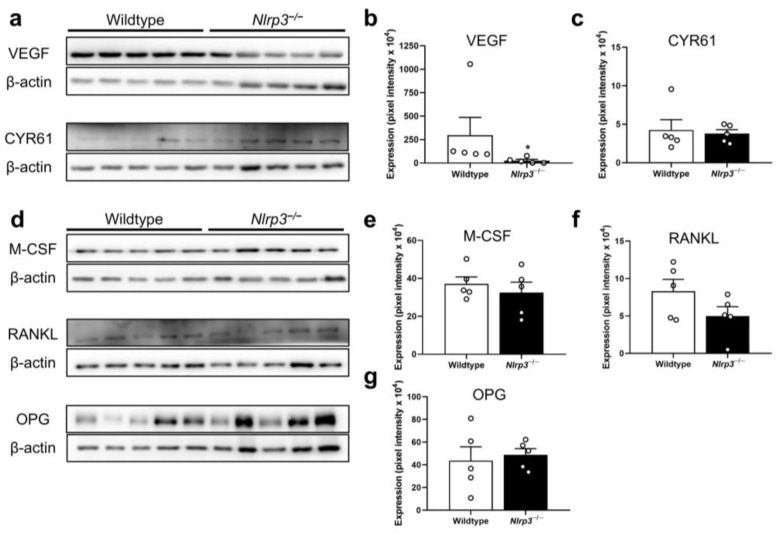
(**a**) Representative Western blots of VEGF, CYR61 and β-actin expression within callus tissue of wildtype and *Nlrp3*^−/−^ mice at 2 weeks after fracture. (**b**,**c**) Expression of VEGF (**b**) and CYR61 (**c**) within callus tissue of wildtype (white bars, *n* = 5) and *Nlrp3*^−/−^ mice (black bars, *n* = 5) (mean ± SEM). * *p* < 0.05 vs. wildtype. (**d**) Representative Western blots of M-CSF, RANKL, OPG and β-actin expression within callus tissue of wildtype and *Nlrp3*^−/−^ mice at 2 weeks after fracture. (**e**–**g**) Expression of M-CSF (**e**), RANKL (**f**) and OPG (**g**) within callus tissue of wildtype (white bars, *n* = 5) and *Nlrp3*^−/−^ mice (black bars, *n* = 5) (mean ± SEM).

The Western blot analyses at 2 weeks after fracture confirmed the lack of NLRP3 expression in the *Nlrp3*^−/−^ mice (*p* = 0.014) (Figure 6a,b). Interestingly, the expression of the absent in melanoma (AIM)2 inflammasome was more than 2-fold higher in the *Nlrp3*^−/−^ mice when compared to the wildtype animals (*p* = 0.015) (Figure 6a,c). However, it should be noted that the expression of this protein was rather heterogenous in the samples from the control group. The expression of the NLR-family CARD-containing protein (NLRC)4 inflammasome did not significantly differ between the two groups (*p* = 0.766) (Figure 6a,d). Moreover, the expression of the pro-inflammatory cytokines IL-1β (*p* = 0.310) and IL-18 (*p* = 0.382), as well as the pro-osteogenic markers, the pro-osteogenic runt-related transcription factor (RUNX)2 (*p* = 0.841) and bone morphogenetic protein (BMP)-4 (*p* = 1.000), showed no significant differences (Figure 6e–j). The expression of the vascular endothelial growth factor (VEGF) was significantly decreased in the *Nlrp3*^−/−^ mice when compared to the wildtype animals (*p* = 0.008) (Figure 7a,b), whereas the expression of the cysteine-rich angiogenic inducer (CYR)61 (*p* = 0.841), the macrophage-colony stimulating factor (M-CSF) (*p* = 0.505), the receptor activator of NF-ĸB ligand (RANKL) (*p* = 0.138) and osteoprotegerin (OPG) (*p* = 0.713) was not affected in the *Nlrp3*^−/−^ mice (Figure 7a,c–g).

## 3. Discussion

In the present study, we investigated, for the first time, the effects of *Nlrp3* deficiency on femoral fracture healing in mice. Surprisingly, we found only marginal differences between the *Nlrp3*^−/−^ and wildtype animals. Moreover, our data demonstrated the unaltered expression of the pro-inflammatory cytokines IL-1β and IL-18, whereas the expression of the AIM2 inflammasome was significantly increased in the *Nlrp3*^−/−^ mice.

An adequate inflammatory response is crucial for bone healing by redirecting mesenchymal stem cells to the fracture site. These cells represent the cell pool for the pro-chondrogenic and pro-osteogenic lineage [13]. It is also well established that the NLRP3 inflammasome is of pivotal importance for inflammatory cytokine production and the regulation of inflammatory processes [9]. However, its role in long bone fracture healing remains elusive. Due to a *Nlrp3* deficiency, the initial inflammatory response may be attenuated, which may delay the recruitment of mesenchymal stem cells to the fracture site. This may lead to the reduced differentiation of the progenitor cells to the osteogenic lineage, such as osteoblasts, and, thus, hamper the process of fracture repair in the early phase of fracture healing. On the other hand, a prolonged or overshooting inflammatory response in the late healing phase may also hamper bone regeneration. In fact, a chronic inflammatory state, which can be observed in the aged, results in reduced bone formation due to increased osteoclast activation and decreased osteoblast formation. Furthermore, a prolonged elevated inflammation is associated with an impaired stem cell function [14,15]. Hence, in contrast to the acute early healing phase, *Nlrp3* deficiency may improve fracture healing in the subacute, late healing period.

Interestingly, Detzen et al. [12] monitored the skeletal development of *Nlrp3*^−/−^ and wildtype mice and demonstrated that the tibia of *Nlrp3*^−/−^ animals showed a lower trabecular bone volume in 4-week-old knockout animals. Notably, the negative effect of *Nlrp3*^−/−^ deficiency appeared to be transitory, as the bone microarchitecture recovered with aging [12]. However, our biomechanical analysis still demonstrated a significantly reduced bending stiffness of the femora of the *Nlrp3*^−/−^ mice when compared to the wildtype animals with an age of 18–22 weeks. These findings indicate that further research regarding the effects of *Nlrp3*^−/−^ deficiency on the long bone microarchitecture and environment is necessary. Alippe et al. [16] provided evidence that *Nlrp3* knockout increases the baseline bone mass in mice and protects from lipopolysaccharide (LPS)-induced calvaria bone resorption. Finally, Li et al. [17] reported that the suppression of the NLRP3 inflammasome improves alveolar bone defect healing in diabetic rats. Additional evidence for the crucial importance of this inflammasome during fracture repair was provided by Sun et al. [18]. They demonstrated impaired bone regeneration in mice with the loss of gasdermin signaling. In addition, Sun et al. [18] showed that the deletion of the IL-1 receptor reproduced the phenotype of gasdermin-deficient mice, resulting in impaired fracture healing. However, these findings are contradictory to those of a previous study of Lange et al. [19], which revealed no significant difference in fracture repair between IL-1 receptor knockout and wildtype animals. Accordingly, further research is required to fully elucidate the complex mechanisms of the inflammasome–interleukin pathways and their influence on fracture repair.

Our µCT data showed only marginally impaired fracture healing in the *Nlrp3*^−/−^ mice. In fact, the expression of markers of bone formation, such as RUNX2 [20,21], BMP-4 [22] and CYR61 [23], as well as the number of osteoclasts and the expression of markers of bone remodeling, i.e., M-CSF [24], RANKL and OPG [25], were not significantly affected in the *Nlrp3*^−/−^ animals. These findings, which are contradictory to those of the study of Li et al. [17], are most likely due to the fact that we analyzed healthy mice, whereas Li et al. [17] performed their analyses on a diabetes model with an overshooting inflammatory response. Hence, inhibition of the NLRP3 inflammasome resulted in reduced pro-inflammatory cytokine production and increased osteogenic gene expression in diabetic rats [17]. Notably, the NLRP3 and AIM2 inflammasomes share a similar pathway by activating pro-caspase 1, thereby processing pro-IL-1β and pro-IL-18 into their active forms. In addition, the inflammasomes cleave gasdermin D, thus stimulating pyroptosis by its N-terminal fragment. Pyroptosis is a lytic form of cell death that allows for the additional release of mature IL-1β and IL-18 from within cells [10]. In our study, we found the unaltered expression of the inflammatory cytokines IL-1β and IL-18. One possible explanation for this finding may be a compensatory upregulation of the AIM2 inflammasome triggered by the *Nlrp3* deficiency. In line with this view, Meng et al. [26] showed in an experimental model of autoimmune uveitis and encephalomyelitis that NLRP3 inhibits the transcription of AIM2 by upregulating the phosphorylation of salt-inducible kinase (SIK) 1 and downregulating the expression of sterol regulatory element-binding transcription factor (SREBF)1. Of interest, SREBF1 is an adipocyte differentiation factor, which produces the protein SREB (SREBP)1. This protein regulates the transcription of over 200 genes involved in the synthesis of fatty acids and triglycerides [27]. Previous reports have demonstrated that NLRP3 and SREBF1 are closely related concerning their roles in liver adipocyte autophagy [28]. The potential interaction of NLRP3, SREBF1 and AIM2 during fracture repair, on the other hand, remains elusive. However, it may be speculated that the NLRP3 and AIM2 inflammasomes mutually regulate and control their expression levels. This hypothesis requires further confirmation by future studies focusing on the potential regulating pathways between these two inflammasomes.

Our immunohistochemical data showed a reduced number of macrophages within the callus tissue of the *Nlrp3*^−/−^ mice at 2 weeks after fracture, whereas the number of neutrophilic granulocytes was significantly increased at this early time point. Notably, granulocytes are the first cells that arrive at a fracture site, where they induce the subsequent recruitment of macrophages by inflammatory mediators, such as IL-6 [6,29]. Therefore, we assume that, in our study, the *Nlrp3* deficiency most probably delayed this cellular inflammatory response. Since macrophages play a major role in triggering the migration of osteogenic precursor cells to a fracture site and induce bone formation, the lower number of macrophages may have also contributed to the reduced trabecular thickness at 2 weeks after fracture in the *Nlrp3*^−/−^ mice.

Moreover, our data revealed the significantly reduced expression of VEGF and the lower number of microvessels within the callus tissue of the *Nlrp3*^−/−^ mice. These results are in line with those of an experimental study of Wang et al. [30], who demonstrated that hypertonic acid alleviates the blood–brain barrier and reduces the infarct volume in astrocytes by inhibiting the NLRP3 inflammasome and, thereby, downregulating VEGF expression. Moreover, the blocking of NLRP3 led to reduced VEGF secretion in a preclinical rat model of diabetic retinopathy [31]. However, the effects of *Nlrp3* deficiency on angiogenesis appear to be controversial. In fact, other reports have shown that the pharmacological blockade of the NLRP3 inflammasome improves the function of endothelial precursor cells, thereby stimulating diabetic wound healing [32]. Moreover, *Nlrp3* deficiency promotes the revascularization of transplanted pancreatic islets, resulting in an improved engraftment in *Nlrp3*^−/−^ mice when compared to wildtype animals. Interestingly, this was not associated with the increased expression of VEGF-A [11]. Therefore, further research is required to fully understand the effects of *Nlrp3* deficiency on angiogenesis and vascularization. Angiogenesis plays a vital role in bone regeneration, since newly formed blood vessels provide an adequate nutrient and oxygen supply and allow for the migration of stem cells to the fracture zone [33,34,35]. Impaired angiogenesis, in turn, delays fracture repair and may even lead to non-union formation [36,37]. Notably, our results demonstrated only a marginally affected fracture healing in the *Nlrp3*^−/−^ mice. Hence, we assume that the angiogenic response in the knockout animals was still sufficient to promote fracture repair.

In summary, we found that *Nlrp*3 deficiency does not markedly affect femoral fracture healing in mice. This is most likely due to the unaltered expression of pro-inflammatory cytokines and pro-osteogenic growth factors. However, the potential compensatory mechanisms between the NLRP3 and AIM2 inflammasomes remain elusive. Hence, future studies should focus on the interaction and potential compensatory mechanisms of these inflammasomes during fracture repair. Thereby, the exact role of these multi-protein complexes during bone regeneration may be elucidated.

## 4. Materials and Methods

### 4.1. Animals

A total number of 23 transgenic *Nlrp3*^−/−^ (B6.129S6-Nlrp3^tm1Bhk^/J) mice [11] and 22 C57BL/6J wildtype mice with an age of 18–22 weeks were used in the present study. The animals were kept on a standard 12 h day/night cycle. Water and standard pellet chow (Altromin, Lage, Germany) were provided ad libitum.

### 4.2. Surgical Procedure

A standardized femoral fracture model was used, as previously described in detail [38]. A closed femoral fracture was induced and stabilized by an intramedullary screw (MouseScrew, AO Development Institute, Davos, Switzerland), providing both fracture reduction and interfragmentary compression [39]. The femora were analyzed 2 (wildtype: *n* = 8; *Nlrp3*^−/−^: *n* = 8) and 5 weeks (wildtype: *n* = 9; *Nlrp3*^−/−^: *n* = 10) after fracture.

### 4.3. X-Ray

To guarantee adequate fracture reduction and to identify possible implant or fracture dislocations, lateral radiographs were performed postoperatively at 2 and 5 weeks before harvesting the femora (MX-20, Faxitron X-ray Corporation, Wheeling, IL, USA).

### 4.4. Biomechanics

After the removal of the soft tissue, the bending stiffness of the harvested fractured and unfractured femora was measured by a non-destructive approach using a 3-point bending device [40]. Loading was stopped for each specimen when the load–displacement curve deviated > 1% from linearity. The bending stiffness (N/mm) was then calculated from the load–displacement diagram, and was given as the absolute bending stiffness (N/mm) as well as the relative bending stiffness as the percent of the corresponding unfractured femurs (%), to account for differences in the individual animals.

### 4.5. µCT

The harvested femora were scanned (Skyscan 1172, Bruker, Billerica, MA, USA) at a spatial resolution of 9 μm with a standardized setup (tube voltage: 50 kV; current: 200 μA; intervals: 0.4°; exposure time: 3500 ms; filter: 0.5 mm aluminum) and analyzed as previously described in detail [41]. The images were stored in 3-dimensional arrays. To express the gray values as mineral content (bone mineral density (BMD)), calcium hydroxyapatite (CaHA) phantom rods with known BMD values (0.250 g and 0.750 g CaHA/cm^3^) were employed for the calibration. The region of interest (ROI) defining the novel bone was contoured manually, excluding any original cortical bone. Thresholding allowed for the differentiation between poorly and highly mineralized bone, as previously described in detail [40].

The following parameters were calculated from the callus ROI for each specimen: highly and poorly mineralized bone volume (mm³), BV/ TV (%)) and trabecular thickness (mm).

### 4.6. Histology and Histomorphometry

The femora were fixed in 4% phosphate-buffered formalin for 24 h, decalcified in 10% ethylene–diamine–tetra-acetic acid (EDTA) solution for 2 weeks and then embedded in paraffin. Longitudinal sections through the femoral axis were cut and stained with Safranin-O for the histomorphometric analyses. At a magnification of 12.5x (Olympus BX60 Microscope, Olympus, Shinjuku, Japan; Zeiss Axio Cam and Axio Vision 3.1, Zeiss, Oberkochen, Germany), the structural indices were calculated according to the recommendations of Gerstenfeld et al. [42]. The following histomorphometric parameters of the callus were evaluated: TOTAr/CAr (%), CgAr/CAr (%), FTAr/CAr (%) and CAr/BDm (mm). Each area and diameter were marked and calculated using ImageJ analysis software (ImageJ 1.54d, NIH, Bethesda, MD, USA).

**Figure 8:**
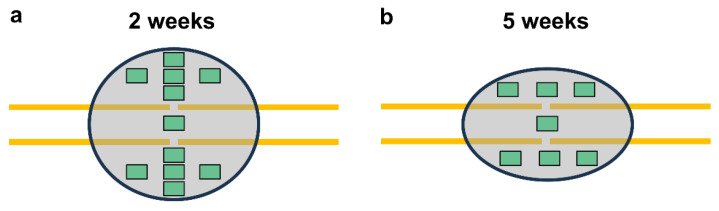
(**a**,**b**) Schematic illustration of the cellular analysis at 2 (**a**) and 5 weeks (**b**) after fracture. One HPF was placed in the central region of the callus (former fracture gap), while five additional HPFs were placed at each site within the periosteal region of the callus in the specimens at 2 weeks after fracture (**a**). Only three HPFs were placed at each site within the periosteal region of the callus at 5 weeks after fracture due to the reduced size of the callus (**b**).

Additionally, the number of TRAP-positive osteoclasts was assessed in the callus tissue at 2 and 5 weeks after fracture healing. For this purpose, the bones were fixed in IHC zinc fixative for 24 h, decalcified in 10% EDTA solution for 2 weeks and then embedded in paraffin. After deparaffinizing, the longitudinal sections were incubated in a mixture of 5 mg naphthol AS-MX phosphate and 11 mg fast red TR salt in 10 mL 0.2 M sodium acetate buffer (pH 5.0) for 1 h at 37 °C. For the microscopic analysis, one high-power field (HPF) was placed in the central region of the callus (former fracture gap), while five additional HPFs were placed at each site within the periosteal region of the callus in the 2-week specimens. In contrast, only 3 additional HPFs were placed at each site within the periosteal region of the callus in the 5-week specimens, due to the reduced size of the callus (Figure 8a,b).

### 4.7. Immunohistochemistry

To analyze the angiogenesis and inflammation within the callus tissue at 2 and 5 weeks after fracture, additional longitudinal tissue sections were cut. For the immunohistochemical detection of microvessels, the sections were stained with a monoclonal rat anti-mouse antibody against endothelial cell marker CD31 (1:100; Dianova, Hamburg, Germany). A goat anti-rat IgG-Alexa Fluor 555 antibody served as the secondary antibody (1:100; Thermo Fisher Scientific Inc., Waltham, MA, USA). The cell nuclei were stained with Hoechst 33342 (2 µg/mL; Sigma-Aldrich, Taufkirchen, Germany). Macrophages and neutrophilic granulocytes within the callus tissue were detected by using a rabbit anti-mouse CD68 antibody (1:300; Abcam, Cambridge, UK) and a rabbit anti-mouse MPO antibody (1:100; Abcam), respectively. A goat anti-rabbit IgG antibody served as the secondary antibody (1:200; Abcam).

The analysis of the CD31-positive microvessels, CD68-positive macrophages and MPO-positive granulocytes per HPF was performed according to the analysis of the TRAP-positive osteoclasts (Figure 8a,b).

### 4.8. Western Blot

Western blotting was performed to analyze the expression of different proteins by their corresponding antibodies within the callus tissue at 2 weeks after fracture (n = 5).

These included the inflammasomes NLRP3 (1:30; Cell Signalling Technology, Danvers, MA, USA), AIM2 (1:30; Cell Signalling Technology) and NLRC4 (1:30; Abcam); the inflammatory cytokines IL-1β (1:30; Cell Signalling Technology) and IL-18 (1:30; Abcam); the pro-osteogenic RUNX2 (1:30; Abcam) and BMP4 (1:30; R&D Systems, Wiesbaden, Germany); the pro-angiogenic VEGF (1:30; Abcam) and CYR61 (1:30; R&D Systems); and markers of bone remodeling, i.e., M-CSF (1:30; Abcam), RANKL (1:30, Proteintech Group, Inc., Rosement, IL, USA) and OPG (1:30; Santa Cruz Biotechnology, Inc., Dallas, TX, USA).

The primary antibodies were followed by their corresponding horseradish peroxidase-conjugated secondary antibodies. Protein expression was visualized by means of luminol-enhanced chemiluminescence after the exposure of the membranes in an Intas ECL Chemocam Imager (Intas Science Imaging Instrument GmbH, Göttingen, Germany) and their normalization to β-actin signals to correct for unequal loading.

### 4.9. Statistical Analysis

All the data are given as the means  ±  SEM. After testing the data for normal distribution (Kolmogorov–Smirnov test) and equal variance (*F*-test), comparisons between the two groups were performed using an unpaired Student’s *t*-test. For the non-parametric data, a Mann–Whitney *U*-test was used. All the statistics were calculated using SigmaPlot 13.0 software (Jandel Corporation, San Rafael, CA, USA). A *p*-value of <0.05 was considered to indicate significant differences.

## Data Availability

The datasets used and/or analyzed during the current study are available from the corresponding author upon reasonable request.
